# On the Role of EFL/ESL Teachers' Emotion Regulation in Students' Academic Engagement

**DOI:** 10.3389/fpsyg.2021.758860

**Published:** 2021-10-06

**Authors:** Fang Wang, Zhi Ye

**Affiliations:** ^1^College of Public Education, Zhejiang Institute of Economics and Trade, Hangzhou, China; ^2^School of Marxism, Zhejiang Police College, Hangzhou, China

**Keywords:** teacher emotion regulation, student academic engagement, EFL/ESL teachers, EFL/ESL, education

## Abstract

Considering the pivotal role of students' academic engagement in their success, discovering the intrinsic and extrinsic factors that inspire students to engage in class activities seems crucial. Notwithstanding, only a few empirical studies have been devoted to teachers' personal factors such as emotion regulation and their predictive function. Further, to our knowledge, no theoretical or systematic review study has been conducted on the association between teacher emotion regulation and student academic engagement. The current review study seeks to fill these lacunas by illustrating English as a foreign language (EFL) or English as a second language (ESL) teachers' emotion regulation and its' capability to enhance students' academic engagement. Using the existing evidence, the power of ESL/EFL teachers' emotion regulation in predicting their pupils' academic engagement was proved. The findings' educational implications are further discussed.

## Introduction

Given the fact that students' disengagement is a major threat to their success (Hiver et al., 2021), fostering students' academic engagement is of high importance for teachers in all instructional-learning contexts, notably ESL/EFL classes. The notion of student engagement is generally conceptualized as “the extent and manner of involvement manifested by learners in relation to academic activities” (Guz and Tetiurka, 2016, p. 136). As a multifaceted construct, student academic engagement covers both emotional and behavioral aspects of students' involvement in performing learning tasks (Christenson et al., 2012). As put forward by Finn and Zimmer (2012), students' academic engagement can remarkably contribute to their increased achievement, mainly due to the fact that engaged students who have positive attitudes toward the learning process commonly put more effort into acquiring the course content. Thus, discovering factors that enhance students' academic engagement seems essential. In line with this premise, the impact of students' personal factors on their academic engagement has been widely studied (e.g., Renninger and Hidi, 2015; Li et al., 2016; Qureshi et al., 2016; Alley, 2019; Khajavy, 2021). Similarly, considerable attention has been paid to the role of teacher interpersonal behaviors (e.g., Estepp and Roberts, 2015; Derakhshan, 2021; Jiang and Zhang, 2021; Xie and Derakhshan, 2021; Zheng, 2021, Dixson et al., 2017). In contrast, the probable effects of teacher personal traits such as emotion regulation has been the focus of less empirical research (e.g., Akbari et al., 2017; Kwon et al., 2017; Burić and Frenzel, 2020).

Teacher emotion regulation as a personal factor pertains to internal and external processes an instructor goes through to assess and manage his/her inner feelings (Thompson et al., 2008). In their study, Cole and Deater-Deckard (2009) conceptualized teacher emotion regulation as “the ability to respond to the ongoing demands of experience with the range of emotions in a manner that is socially tolerable and sufficiently flexible to permit spontaneous reaction as well as the ability to delay spontaneous reactions as needed” (p. 1327). Regulating their emotions, teachers can create a desirable learning environment (Shao et al., 2020; Fathi et al., 2021), which is fundamental for involving pupils in the learning process.

Despite the facilitative function that teacher emotion regulation may serve in engaging students, a few studies (Akbari et al., 2017; Kwon et al., 2017; Burić and Frenzel, 2020) have empirically explored the impact of teacher emotion regulation on ESL/EFL students' academic engagement. Further, no theoretical review study has been conducted on the association between these variables. Hence, in this study, the researcher aims to explicate different conceptualizations of teacher emotion regulation and student academic engagement, their theoretical underpinnings, and their interrelationships.

### Teacher Emotion Regulation

The construct of emotion regulation is generally conceptualized as “individuals' ability to respond to the ongoing demands of experience with the range of emotions in a manner that is socially tolerable and sufficiently flexible to permit spontaneous reaction” (Cole et al., 1994, p. 76). More specifically, teacher emotion regulation is defined as the ability of instructors to manage their emotional experiences in classroom contexts (Fried, 2011). Emotion regulation strategies that teachers utilize before and after the generation of emotions are called *response-focused strategies* and *antecedent-focused strategies*, respectively (Greenier et al., 2021). Among various response-focused and antecedent-focused strategies, one can refer to expressive suppression and cognitive reappraisal as two main strategies that teachers frequently employ to navigate their feelings. Cognitive reappraisal is described as one's endeavor to reframe an emotional event or experience in such a manner that modifies its underlying meaning and impression (Troy et al., 2018). Expressive suppression is also defined as individuals' effort to hide, impede, or restrict emotion-expressive behavior (Cutuli, 2014).

### Student Academic Engagement

Student academic engagement is primarily defined as “the quality of students' participation or connection with the educational endeavor and hence with activities, values, individuals, aims, and place that comprise it” (Skinner et al., 2009, p. 496). This concept is further conceptualized as students' cognitive, emotional, and behavioral investment in learning academic content (Archambault et al., 2009). In line with this conceptualization, Reeve and Tseng (2011) divided the construct of student academic engagement into four components of “*behavioral engagement*,” “*cognitive engagement*,” “*emotional engagement*,” and “*agentive engagement*.” Behavioral engagement, as the first component, refers to students' amount of effort, persistence, and attention in doing learning activities. As the second component, cognitive engagement pertains to students' adoption of advanced learning techniques and self-regulation strategies. Emotional engagement also relates to students' interest and passion for acquiring course content. Agentive engagement as the last component of student academic engagement refers to “pupils' positive contribution to the flow of instruction” (Alrashidi et al., 2016, p. 43).

### The Positive Connection Between ESL/EFL Teachers' Emotion Regulation and Their Students' Academic Engagement

To illuminate the importance of ESL/EFL teachers' emotion regulation in fostering students' academic engagement, Dewaele and Li (2020) stated that teachers who are able to control and navigate their emotions can create a positive atmosphere which is crucial for raising students' learning engagement. In this regard, Kwon et al. (2017) also postulated that the manner in which teachers control or express their feelings can enormously affect students' academic functioning, notably academic engagement. To them, those who do not express severe emotional reactions to students' misbehaviors are more successful in motivating pupils to participate in class activities. Similarly, in light of positive psychology core assumptions, Wang et al. (2021) proposed that the emotion regulation strategies that instructors utilize to down-regulate their undesirable feelings enable them to establish close relationships with their students. Those students who have favorable relations with their instructors are more motivated to engage in learning activities (Reyes et al., 2012; Quin, 2017; Martin and Collie, 2019; Xie and Derakhshan, 2021).

## Empirical Studies

Given the undeniable role of ESL/EFL students' academic engagement in their success (Hiver et al., 2021), a large amount of study has been devoted to this construct and its predictors. Nevertheless, limited attention has been paid to the predictive power of teacher personal factors such as emotion regulation (Akbari et al., 2017; Kwon et al., 2017; Burić and Frenzel, 2020). Kwon et al., 2017, for instance, explored the association between teacher emotion regulation and students' engagement. Distributing two validated questionnaires among participants, their perspectives on the impact of teacher emotion regulation on students' academic success were obtained. The inspection of the correlations portrayed a favorable relation between teacher emotion regulation and students' learning engagement. In a qualitative study, Akbari et al. (2017) also investigated the positive consequences of teacher emotion regulation for EFL students' academic behaviors. In doing so, some semi-structured interview sessions were performed. Analyzing participants' answers to interview questions, the researchers reported that the emotion regulation strategies that teachers utilize in EFL classes can dramatically affect student academic behaviors, notably academic engagement. In a similar vein, Burić and Frenzel (2020) also examined the role of instructors' emotion regulation in their pupils' academic engagement. To gather data, Teacher Emotion Regulation Scale (TERS) and Student Engagement in Schools Questionnaire (SESQ) were given to participants. Analyzing participants' responses to the questionnaires, the researchers discovered a positive connection between teachers' emotion regulation and students' engagement. That is, students viewed teacher emotion regulation as a strong antecedent of their engagement.

## Conclusion and Pedagogical Implications

In the current review study, the variables of teacher emotion regulation and student academic engagement were fully described. Further, the favorable association between these constructs was clarified. The existing literature was then reviewed to prove the capability of teacher emotion regulation in fostering students' academic engagement. Considering the empirical and theoretical evidence, it is logical to conclude that teacher emotion regulation as an important personal trait can drastically influence students' propensity to actively engage in the learning experience. This finding seems to be illuminative for teacher trainers who are expected to give teachers adequate instructions on how to control and navigate their emotions in instructional-learning settings. It is mainly due to the fact that in order to motivate students to become involved in the learning process, teachers should be able to control their negative reactions to students' misconducts (Kwon et al., 2017). Besides, both EFL and ESL teachers can also benefit from the findings of this study. As put forward by Martin and Collie (2019), a positive learning environment is vital for raising students' engagement. To them, teachers can create such desirable atmosphere through controlling their emotions. Accordingly, those teachers who aspire to improve their pupils' academic engagement should regulate their emotions in classroom contexts. Given the paucity of research on this topic, more empirical and theoretical investigations are required to delve into the association of teacher emotion regulation and student academic engagement. Due to the importance of emotion regulation in academic contexts, some cross-cultural studies are also suggested to be conducted on the role of this construct and other variables related to positive psychology. Besides, with the unprecedented outbreak of COVID-19, it stands to reason to delve into how such dynamic negative factors as boredom (Li and Dewaele, 2020; Derakhshan et al., 2021; Kruk, 2021; Wang and Derakhshan, 2021) and burnout (Fathi et al., 2021) can affect students' engagement and learning.

## Author Contributions

FW and ZY have approved it for submission to Frontiers in Psychology. All authors contributed to the article and approved the submitted version.

## Funding

The review was supported by the Fundamental Research Funds for the Provincial Universities, Zhejiang Institute of Economics and Trade (Grant Number: 21YQ08): The relationship and interaction mechanism between the short video social network and depression for college students.

## Conflict of Interest

The authors declare that the research was conducted in the absence of any commercial or financial relationships that could be construed as a potential conflict of interest.

## Publisher's Note

All claims expressed in this article are solely those of the authors and do not necessarily represent those of their affiliated organizations, or those of the publisher, the editors and the reviewers. Any product that may be evaluated in this article, or claim that may be made by its manufacturer, is not guaranteed or endorsed by the publisher.
